# Supplementary material for: Allostatic load and its determinants in a German sample—Results from the Carla cohort
Source: PLoS One. 2025 Apr 24;20(4):e0321178. doi: 10.1371/journal.pone.0321178 (PMC12021213; doi:10.1371/journal.pone.0321178)
Supplement: S1 Table — (DOCX) [file pone.0321178.s001.docx]

**Supporting information**

| **S1 Table: Descriptive summary for the whole sample** | | | | |
| --- | --- | --- | --- | --- |
| **Characteristics at baseline** |  | **Men**  **n=967** | **Women**  **n=812** | **All**  **n=1779** |
| Mean AL score | Baseline | -0.41 | -1.14 | **-0.74** |
|  | First follow-up | -0.92 | -1.47 | **-1.17** |
|  | Third follow-up | -1.59 | -2.01 | **-1.79** |
| Mean age in years |  | 64.9 | 63.8 | **64.4** |
| Income^a^ | low | 63.2% | 72.0% | **67.2%** |
|  | high | 36.8% | 28.0% | **32.8%** |
| Education^b^ | low | 39.4% | 42.2% | **40.7%** |
|  | high | 60.6% | 57.8% | **59.3%** |
| Profession^c^ | low | 59.0% | 84.0% | **70.4%** |
|  | high | 41.0% | 16.0% | **29.6%** |
| number of children (mean) |  | 1.9 | 1.8 | **1.8** |
| marital status | divorced | 8.6% | 12.6% | **10.4%** |
|  | married | 80.9% | 58.3% | **70.5%** |
|  | single | 3.1% | 6.3% | **4.6%** |
|  | widowed | 7.5% | 22.9% | **14.5%** |
| use of nicotin | current smoker | 23.3% | 14.7% | **19.3%** |
|  | ex smoker | 51.4% | 17.2% | **35.8%** |
|  | never smoked | 25.4% | 68.1% | **44.9%** |
| alcohol consumption | g/day (mean) | 17.5 | 4.1 | **11.4** |
| Pack-years of tabacco | (mean) | 15.6 | 4.3 | **10.4** |
| sport | hours/week (mean) | 0.7 | 0.8 | **0.8** |
| antihypertensive medication | yes | 55.1% | 55.7% | **55.4%** |
| lipid lowering medication | yes | 17.3% | 15.8% | **16.6%** |
| antidiabetic medication | yes | 12.9% | 11.5% | **12.3%** |
|  | | | | |

^a^ Net equivalent income per person < 1500€, ^b^ secondary school or lower, ^c^ unskilled worker, semiskilled, skilled worker, farmer, foreman, assisting family, simple employee or lower civ. Servant, employee foreman, qualified employee, middle civil servant
