# Supplementary material for: Allostatic load and its determinants in a German sample—Results from the Carla cohort
Source: PLoS One. 2025 Apr 24;20(4):e0321178. doi: 10.1371/journal.pone.0321178 (PMC12021213; doi:10.1371/journal.pone.0321178)
Supplement: S2 Table — (DOCX) [file pone.0321178.s002.docx]

**S2 Table: Counts of missing data points for biomarkers at baseline and follow-up examinations**

| ***Biomarker*** | *BMI^a^* | *Waist^b^* | *CRP^c^* | *Triglycerides* | *LDL^d^* | *HDL^e^* | *HbA1_c_^f^* | *RR_sys_^g^* | *RR_dia_^h^* |
| --- | --- | --- | --- | --- | --- | --- | --- | --- | --- |
| ***Carla-0*** | 0/473 | 0/473 | 27/473 | 2/473 | 2/473 | 2/473 | 2/473 | 0/473 | 0/473 |
| ***Carla-1*** | 0/473 | 0/473 | 1/473 | 1/473 | 1/473 | 1/473 | 1/473 | 0/473 | 0/473 |
| ***Carla-3*** | 1/473 | 1/473 | 32/473 | 33/473 | 30/473 | 30/473 | 32/473 | 5/473 | 5/473 |

*^a^body mass index, ^b^waist circumference, ^c^c-reactive protein, ^d^low-density lipoprotein, ^e^high-density lipoprotein, ^f^glycated hemoglobin, ^g^systolic blood pressure, ^h^diastolic blood pressure*
