# Supplementary material for: Allostatic load and its determinants in a German sample—Results from the Carla cohort
Source: PLoS One. 2025 Apr 24;20(4):e0321178. doi: 10.1371/journal.pone.0321178 (PMC12021213; doi:10.1371/journal.pone.0321178)
Supplement: S3 Table — (DOCX) [file pone.0321178.s003.docx]

| **S3 Table: Comparison of different calculation methods for AL scores Mean [95% CI]** | | | |
| --- | --- | --- | --- |
|  | ***Carla-0*** | ***Carla-1*** | ***Carla-3*** |
| ***Cumulative z-scores*** | -1.66  [-1.98; -1.34] | -2.14  [-2.45; -1.83] | -2.30  [-2.57; -2.02] |
| ***Sum score^a^*** | 3.31  [3.15; 3.56] | 2.93  [2.78; 3.08] | 2.89  [2.76; 3.02] |
| ***Sum score^b^*** | 3.43  [3.27; 3.59] | 3.18  [3.03; 3.32] | 3.17  [3.03; 3.28] |

^a^without consideration for medication use, ^b^with consideration for medication use
