# Supplementary material for: Allostatic load and its determinants in a German sample—Results from the Carla cohort
Source: PLoS One. 2025 Apr 24;20(4):e0321178. doi: 10.1371/journal.pone.0321178 (PMC12021213; doi:10.1371/journal.pone.0321178)
Supplement: S4 Table — (DOCX) [file pone.0321178.s004.docx]

| **S4 Table: characteristics of time-variant variables at first follow-up (sub-cohort)** | | | |
| --- | --- | --- | --- |
| **Characteristics** | Men (n= 245) | Women (n= 228) | Total (n= 473) |
| divorced | 9.7% | 10.8% | 10.3% |
| married | 83.7% | 68.5% | 76.3% |
| single | 3.3% | 6.0% | 4.6% |
| widowed | 3.3% | 14.7% | 8.8% |
|  |  |  |  |
|  |  |  |  |
| current smoker | 13.9% | 9.1% | 11.5% |
| ex smoker | 50.6% | 26.3% | 38.8% |
| never smoker | 33.0% | 62.9% | 47.6% |
| occasional smoker | 2.5% | 1.7% | 2.1% |
|  |  |  |  |
| alcohol in g/day (mean) | 15.7 | 4.7 | 10.4 |
| packyears of tobacco (mean) | 11.5 | 4.1 | 7.9 |
| hours of sport/week (mean) | 1.7 | 1.9 | 1.8 |
| antihypertensive medication - yes | 53.5% | 52.2% | 52.3% |
| antihypertensive medication - no | 46.5% | 47.8% | 47.7% |
| lipid lowering medication – yes | 18.4% | 14.7% | 16.6% |
| lipid lowering medication – no | 81.6% | 85.3% | 83.4% |
| antidiabetic medication – yes | 6.9% | 6.5% | 6.7% |
| antidiabetic medication – no | 93.1% | 93.5% | 93.3% |
