# Supplementary material for: Allostatic load and its determinants in a German sample—Results from the Carla cohort
Source: PLoS One. 2025 Apr 24;20(4):e0321178. doi: 10.1371/journal.pone.0321178 (PMC12021213; doi:10.1371/journal.pone.0321178)
Supplement: S5 Table — (DOCX) [file pone.0321178.s005.docx]

| **S5 Table: AL scores stratified by age cohorts for men** | | | | | | | | | | | |
| --- | --- | --- | --- | --- | --- | --- | --- | --- | --- | --- | --- |
| *Age* | *< 55* | | *55-< 60* | *60-< 65* | | *65-< 70* | | *70-< 75* | | *75-< 80* | |
| *n* | *102* | | *50* | *49* | | *29* | | *11* | | *4* | |
| **AL score Mean [95% CI]** | | | | | | | | | | | |
| ***Carla-0*** | -1.13  [-1.84; -0.42] | -0.71  [-1.59; 0.16] | | | -0.09  [-1.07; 0.89] | | -0.93  [-1.99; 0.14] | | -2.31  [-3.92; -0.69] | | -3.38  [-5.77; -0.99] |
| ***Carla-1*** | -1.72  [-2.42; -1.01] | -1.33  [-2.15; -0.51] | | | -0.80  [-1.66; 0.06] | | -2.42  [-3.52; -1.31] | | -2.75  [-4.46; -1.03] | | -4.38  [-5.44; -3.32] |
| ***Carla-3*** | -1.54  [-2.14; -0.93] | -1.94  [-2.68; -1.20] | | | -2.16  [-3.05; -1.26] | | -3.30  [-4.35; -2.24] | | -3.20  [-5.60; -0.80] | | -5.21  [-7.42; -2.99] |
