# Supplementary material for: Allostatic load and its determinants in a German sample—Results from the Carla cohort
Source: PLoS One. 2025 Apr 24;20(4):e0321178. doi: 10.1371/journal.pone.0321178 (PMC12021213; doi:10.1371/journal.pone.0321178)
Supplement: S6 Table — (DOCX) [file pone.0321178.s006.docx]

| **S6 Table: AL scores stratified by age cohorts for women** | | | | | | |
| --- | --- | --- | --- | --- | --- | --- |
| *Age* | *< 55* | *55-< 60* | *60-< 65* | *65-< 70* | *70-< 75* | *75-< 80* |
| *n* | 97 | 44 | 49 | 28 | 5 | 5 |
| **AL score Mean [95% CI]** | | | | | | |
| ***Carla-0*** | -3.47  [-4.24; -2.71] | -1.86  [-2.73; -0.99] | -2.50  [-3.45; -1.56] | -0.32  [-1.58; 0.95] | -0.70  [-4.21; 2.82] | -1.83  [-4.49; 0.83] |
| ***Carla-1*** | -3.89  [-4.60; -3.18] | -2.12  [-3.18; -1.06] | -2.53  [-3.52; -1.54] | -0.55  [-1.67; 0.57] | -0.29  [-3.68; 3.09] | -0.38  [-3.47; 2.70] |
| ***Carla-3*** | -2.94  [-3.64; -2.24] | -2.61  [-3.35; -1.88] | -2.37  [3.29; 1.45] | -1.59  [-2.43; -0.75] | 0.36  [-1.79; 2.50] | -3.29  [-7.18; 0.60] |
